# Differential Fatigue Profile in Patients with Post-COVID Condition, Fibromyalgia, and Multiple Sclerosis

**DOI:** 10.3390/jcm14030952

**Published:** 2025-02-02

**Authors:** Silvia Oliver-Mas, Jordi A. Matias-Guiu, Cristina Delgado-Alonso, Constanza Cuevas, José Manuel Alcalá Ramírez del Puerto, Juan Ignacio López-Carbonero, Jorge Matias-Guiu, Maria Diez-Cirarda

**Affiliations:** Department of Neurology, Hospital Clínico San Carlos, San Carlos Health Research Institute (IdISSC), Universidad Complutense de Madrid, 28040 Madrid, Spain

**Keywords:** fatigue, assessment, fibromyalgia, multiple sclerosis, Post-COVID Condition, FICS scale

## Abstract

**Background/Objectives:** Fatigue is a prevalent and debilitating symptom in Post-COVID Condition (PCC), fibromyalgia, and multiple sclerosis (MS). Although these conditions share clinical similarities, the underlying mechanisms of fatigue across these conditions may differ and remain poorly understood. This study aimed to compare the intensity and characteristics of fatigue in these three conditions to identify shared and distinct features. **Methods:** We conducted a cross-sectional study involving 429 participants: 219 with PCC, 112 with fibromyalgia, and 98 with MS. Participants completed a questionnaire specifically developed for the study via the Google Forms platform. This questionnaire was developed by a group of professionals in the hospital specializing in fatigue related to these three conditions, in collaboration with expert patients. The questionnaire was reported following the Checklist for Reporting Results of Internet E-Surveys (CHERRIES) recommendations. **Results:** Fatigue intensity was significantly higher in PCC and fibromyalgia compared to MS. Some differences in fatigue characteristics were observed: MS patients reported more fatigue in response to heat and a greater impact of mood on fatigue. Furthermore, delayed fatigue and reduced benefits from rest were more pronounced in both PCC and fibromyalgia. No significant differences were found regarding cognitive fatigue or difficulties in predicting the ability to perform activities. **Conclusions:** These results underscore some clinical characteristics in the intensity and quality of fatigue across PCC, fibromyalgia, and MS. These findings could suggest different mechanisms in the pathophysiology of the fatigue. Our study underscores the need for tailored diagnostic tools and interventions in managing fatigue in these three conditions.

## 1. Introduction

Fatigue is a persistent feeling of tiredness or lack of energy, which may be categorized as either peripheral or central. Peripheral fatigue refers to muscle fatigue, which results from muscle and neuromuscular junction disorders that make it difficult to move or perform activities in daily living. However, central fatigue is characterized by a sense of exhaustion that hinders the initiation and continuation of activities, even during rest [1]. Furthermore, central fatigue often includes a cognitive aspect known as cognitive fatigue, characterized by reduced effort or performance during mentally demanding tasks [2]. Central fatigue is also associated with other symptoms such as pain, sleep disorders, and affective alterations [3].

Fatigue is a common symptom in many medical conditions, including cancer [4], infections [5], or neurological diseases such as Parkinson’s disease [6], as well as in mental health conditions such as depression [7]. The striatum, dorsolateral prefrontal cortex, insula, and ventromedial prefrontal cortex have been identified as central hubs of the fatigue network in functional connectivity studies [8]. However, the manifestation of fatigue across different populations is still poorly understood.

Post-COVID Condition (PCC), fibromyalgia, and multiple sclerosis (MS) share some clinical characteristics, which have been related to the dysfunction of the central nervous system (CNS), including inflammatory implications [9,10,11]. However, the medical context of these conditions differs: PCC involves individuals who have a history of SARS-CoV-2 infection, with symptoms persisting for more than three months [12]; fibromyalgia is a chronic condition characterized primarily by widespread pain among other symptoms [13]; and MS is a chronic autoimmune disease of the central nervous system marked by inflammation, demyelination, and axonal damage [14]. One common clinical characteristic of these three diseases is that fatigue is one of the most disabling symptoms [13,15,16], which impacts daily life activities, work, and social life [17,18,19], leading to significant socioeconomic consequences [18,20,21].

However, fatigue is still predominantly studied within the context of each individual disease. Comparative studies are lacking, and they could be useful to better understand the experience of fatigue and its impact on daily life across these three different diseases. Given the subjective nature of fatigue as a symptom, self-report questionnaires are likely the most reliable for its assessment [22,23]. Currently, the most commonly used scales are the Modified Impact Fatigue Scale (MFIS) [24] and the Fatigue Severity Scale (FSS) [25]. Considering the impact of fatigue, it is essential that we conduct a detailed evaluation that considers the different mechanisms involved (e.g., central or peripheral), as well as other symptoms, such as pain or depression, that may influence fatigue. We hypothesized that some differences in the characteristics of the fatigue and associated symptoms could be present in PCC, fibromyalgia, and MS. The identification of differential characteristics in the fatigue and other symptoms could be useful for diagnostic purposes for each entity, but also to develop specific interventions in each case. Therefore, our study aimed to compare the characteristics of fatigue among PCC, fibromyalgia, and MS by applying a detailed questionnaire specifically designed to evaluate the intensity and characteristics of the fatigue in these three conditions.

## 2. Materials and Methods

### 2.1. Design and Participants

This study was a cross-sectional comparative study which involved 429 participants: 219 patients with PCC, 112 patients with fibromyalgia, and 98 patients diagnosed with MS.

The mean age of participants was 49.81 ± 8.24 years in the PCC group, 52.56 ± 7.88 years in fibromyalgia, and 48.15 ± 9.21 years in MS (*p* = 0.001). Regarding sex, there were 185 (84.5%) women with PCC, 107 (95.6%) women with fibromyalgia, and 69 (70.4%) with MS (*p* < 0.001). Participants were recruited at different years of evolution since the infection (PCC) or disease diagnosis (fibromyalgia and MS); patients with PCC had 3.16 ± 0.94 years of evolution, patients with fibromyalgia had 8.67 ± 8.00 years of evolution, and patients with MS had 11.41 ± 8.69 years of evolution (*p* < 0.001) (Table 1).

Most participants showed an impairment on the MFIS (MFIS ≥ 38) [26], observed in 218 PCC participants (99.5%), 111 fibromyalgia patients (99.1%), and 88 MS participants (90%). Significant differences were found across PCC, fibromyalgia, and MS participants (*p* < 0.001). Moreover, we found significant differences among groups regarding the percentage of patients smoking tobacco and the percentage of patients with a diagnosis of autoimmune disease, showing PCC patients with a reduced percentage compared to fibromyalgia and MS in both cases, and fibromyalgia patients also revealed significantly increased autoimmune diagnosis compared to MS. Demographic and clinical characteristics are described in Table 1.

The inclusion criterion was the medical diagnosis of PCC, fibromyalgia, or MS. Patients with comorbidity across the three conditions (e.g., diagnosis of two diseases, such as fibromyalgia and MS) were excluded.

The study was reported following the “Checklist for Reporting Results of Internet E-Surveys” (CHERRIES) recommendations [27].

### 2.2. Questionnaire

The Fatigue Intensity and Characteristics Scale (FICS) (Supplementary Materials) aimed to describe the intensity and characteristics of fatigue in patients with PCC, fibromyalgia, and MS. The ethics committee from Hospital Clínico San Carlos approved the study on 11 May 2023 (code 23/220-E). Moreover, participants were informed about the purpose of the questionnaire and were required to give signed consent before participating. To ensure participant privacy, data were collected anonymously.

This questionnaire was developed by a committee of neurologists and psychologists with expertise in fatigue symptoms in patients with PCC, fibromyalgia, and MS. Additionally, five expert patients participated in the development to understand the patient experience better and ensure that the language used in the questionnaire was understandable for the participants. The questionnaire was open only to participants with the conditions we mentioned before. Between April and September 2024, we contacted patients’ associations who then distributed the Google Forms link to the participants via e-mail. Participants could complete the questionnaire by clicking the link to start. The questionnaire consisted of seven pages: the first page provided information about the study, followed by six sections on the subsequent pages. The completion of the questionnaire was voluntary without incentive.

The first section of the FICS questionnaire collected demographic and clinical data from the participants, comprising 29 items (Section A), which included multiple-choice options and open-ended questions. The second section gathered data on COVID-19 infections and vaccination, consisting of 61 items that included both multiple-choice options and open-ended questions (Section B). Moreover, the third section comprised the Intensity Scale, which included 18 questions assessing the intensity of fatigue, to obtain a more detailed description (Section C). The fourth section, the Characteristics Scale, was designed to obtain information on symptoms related to the characteristics of fatigue and consisted of 32 items (Section D). The questions on the third scale (Intensity Scale) and fourth scale (Characteristics Scale) were multiple-choice with a five-point Likert Scale: “0 = strongly disagree”, “1 = slightly agree”, “2 = moderately agree”, “3 = quite agree”, and “4 = strongly agree”.

In addition, the fifth section was specific to fibromyalgia and MS questionnaires, providing more detailed information about these conditions. The fibromyalgia questionnaire included six items (Section E) while the MS questionnaire contained seven items (Section F) comprising both multiple-choice questions and open-ended questions.

Finally, participants responded to MFIS questionnaire which contained 21 items [24].

We estimated that the questionnaire would take approximately 20 min to complete. In addition, the participants’ responses were automatically saved by Google Forms and e-mail addresses were reviewed to ensure that none were duplicated.

### 2.3. Sample Size Estimation

The sample size was determined based on the subject-to-item ratio [28]. A minimum ratio of 10 subjects per item was selected based on previous recommendations for ensuring adequate power in psychometric testing [29,30]. The study included 429 participants, with 23 subjects-per-item in the Intensity Scale and 13 subjects-per-item in the Characteristics Scale.

### 2.4. Statistical Analysis

Statistical analyses were performed using the Statistical Package for the Social Sciences (SPSS) v. 22.0. To assess the internal consistency of the FICS, we calculated Cronbach’s alpha. Concurrent validity was evaluated by comparing the FICS Intensity Scale with the total score of the MFIS. Differences between groups were performed with the analysis of variance (ANOVA) or χ^2^ test when appropriate, due to the large sample size, and statistical significance was set at *p* < 0.05.

The comparisons across the three groups were conducted using ANOVA followed by Tukey’s post hoc test. Bonferroni correction was applied for ANOVA analysis in the Intensity Scale *p* < 0.0027 and Characteristics Scale *p* < 0.0015, corrected by the number of items in each scale.

Finally, we sought to control for the effect of age on the Intensity and Characteristics Scales due to the differences among groups and the reported relationship with fatigue [31,32,33], and control for fatigue severity (measured with the MFIS total score) on the Characteristics Scale, in order to assess the differences in qualitative fatigue without the influence of fatigue severity; the results of the fatigue Intensity Scale were covaried by age and the results of the fatigue Characteristics Scale were adjusted for age and fatigue severity (MFIS total score), including only patients with significant fatigue (MFIS ≥ 38) [26] in all three conditions.

## 3. Results

### 3.1. Internal Consistency of the FICS Questionnaire

The Intensity Scale (Section C) obtained an internal consistency of α = 0.926, which could be interpreted as a high internal consistency. This was expected, as all the items on the scale are designed to align in the same direction, either indicating a high or low intensity.

Moreover, the Characteristics Scale (Section D) obtained an internal consistency of α = 0.681. This internal consistency can be interpreted as “acceptable” because it is a preliminary scale. This was also expected: because of the heterogeneity of the Characteristics Scale, not all the items should go in the same direction, taking into account the fact that it included items for three different pathologies.

Regarding the concurrent validity, the Intensity Scale of the FICS showed a strong positive and significant correlation with the MFIS total score (r = 0.657; *p* < 0.001).

### 3.2. Comparisons Across PCC, Fibromyalgia, and MS in the Intensity Scale

In the Intensity Scale, which consists of 18 items, there was a group effect in all items except for C10 and C11. These items refer to the participant’s difficulty in starting and finishing things because he/she knows he/she will not be able to finish them (Table 2).

The post hoc analysis showed that PCC and fibromyalgia showed significantly higher scores than MS in items that refer to tiredness and lack of energy (C1, C3, and C12), a need to sleep more hours (C2), muscular strength (C5), pain (C6 and C7), concentration difficulties (C8), and the impact of fatigue on physical activity (C14) (Table 2).

However, only PCC scored significantly higher than MS in item C15, which refers to the impact of fatigue in a patient’s life, as well as on item C16, which refers to feeling bad about being tired (Table 2).

Furthermore, only fibromyalgia showed significantly higher scores than MS in items that refer to apathy (C4 and C9), the persistent feeling of tiredness regardless of physical activity (C13), and the interference of fatigue in sexual desire fatigue (C18) (Table 2).

No statistically significant differences were observed across PCC and fibromyalgia in any of the items of the Intensity Scale (Table 2).

Finally, after performing an ANCOVA controlling for age, all the above items remained statistically significant except C4 (Supplementary Table S1).

### 3.3. Comparisons Across PCC, Fibromyalgia, and MS in the Characteristics Scale

Regarding the Characteristics Scale, which comprises 32 items, we found significant differences between groups in 19/32 items. Specifically, MS scored higher than PCC and fibromyalgia in the negative impact of mood on fatigue (D12) and in fatigue that worsens with heat (D4), as well as in fatigue improvements after rest (D7, D13, D14, D15, and D27), and less fatigue caused by motivational activities (D32) (Table 3). Conversely, fibromyalgia scored higher than MS and PCC in fatigue accompanied by muscle pain (D22).

Additionally, PCC and fibromyalgia scored higher than MS in items related to fatigue appearing later after exertion (D16, D18, and D19), fatigue accompanied by headaches and joint pain (D25 and D26), and episodes of worsening fatigue (D30) (Table 3). Moreover, differences across PCC and MS were found in D5, which refers to fatigue worsening with the cold, with PCC scoring higher; and in D11, which relates to the need to take more breaks during physical exercise, with MS scoring higher than PCC (Table 3).

On the contrary, we did not find significant differences in those items related to fatigue variations throughout the day (D2), feeling motivated despite fatigue (D3), the need to take breaks after physical or cognitive activity (D6, D8, and D9), energy depletion after demanding activities (D10 and D17), changes in fatigue after meals (D20 and D21), physical fatigue being worse than mental fatigue (D23), and difficulty predicting when fatigue will occur (D28, D29, and D31) (Table 3).

Significant differences between groups in the Characteristics Scale items remained statistically significant when controlling by age (except D17) and by age and MFIS (except D5, D16, D17, D18, and D19) (Table 4).

## 4. Discussion

**Figure 1:**
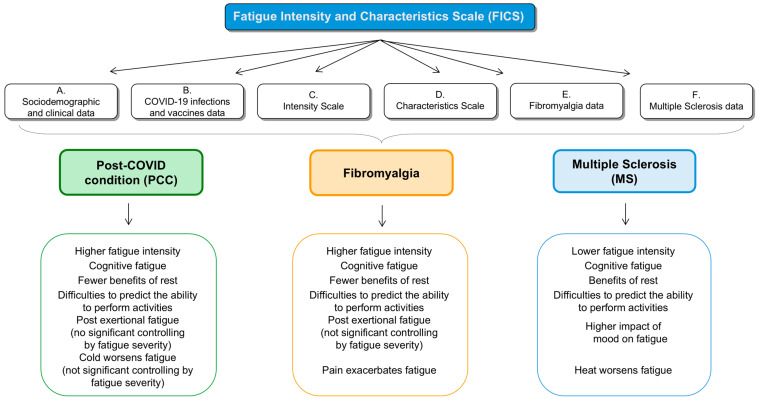
Fatigue Intensity and Characteristics Scale (FICS) and main findings.

Fatigue is a prevalent symptom across numerous medical conditions; yet, its underlying mechanisms remain poorly understood. In this study, we assessed the differences in fatigue among patients with PCC, fibromyalgia, and MS to understand better the clinical characteristics of the fatigue associated with these conditions. An extensive questionnaire was administered to all participants, revealing that fatigue was consistently reported in all three groups, which concurs with previous studies [15,16,34]. Our findings revealed both similarities and significant differences in the intensity and characteristics of fatigue among the three groups (Figure 1).

The three conditions reported characteristics of central fatigue; all three groups reported difficulties in predicting whether they would be able to initiate, sustain, and complete activities throughout the day. Moreover, all three groups experienced a need for rest during cognitively demanding activities, such as conversations, reflecting cognitive fatigue [2], a phenomenon reported in the literature on these three conditions [11,35,36]. However, our results indicated that physical fatigue was perceived as even more debilitating than cognitive fatigue by all groups. Moreover, patients with MS showed a greater impact of mood on fatigue, suggesting a connection between fatigue and affective disorders, which is also characteristic of central fatigue [37].

Additionally, the role of the CNS in fatigue has been previously described in the literature for all three conditions. In MS patients, fatigue is primarily mediated by the CNS, including cortical–striatal dysfunction and disruptions in the glutamatergic and dopaminergic system [38]. Similarly, in PCC, fatigue has been linked to structural and functional connectivity, particularly in the frontal temporal and cerebellar areas [39]. Additionally, in patients with fibromyalgia, symptoms such as fatigue or pain have been characterized as central sensitization [40,41].

However, it seems that patients with PCC and fibromyalgia experience additional symptoms that may exacerbate the intensity of fatigue, which could explain why fatigue is more intense in these two groups. In PCC, fatigue is also linked to peripheral mechanisms including structural and functional muscle damage [42,43]. This aligns with the results of the present study because PCC reported greater muscle weakness and muscle pain. Likewise, patients with fibromyalgia report that fatigue is often accompanied by muscle pain. This interplay suggests that pain may exacerbate the sensation of fatigue in fibromyalgia. Previous research has also highlighted a strong connection between fatigue and pain in fibromyalgia [44]. Moreover, the higher intensity of fatigue in PCC and fibromyalgia could also explain why these two groups experience fewer benefits of rest than MS and are more likely to develop delayed fatigue after physical or cognitive activity, a phenomenon previously linked to fatigue intensity [45]. In this regard, we found that post-exertional malaise was more severe in PCC and fibromyalgia than MS, but not after controlling for fatigue severity. This suggests that this symptom may be a marker of severity, but it is not specific to any disorder.

Furthermore, fatigue in MS patients worsens with heat exposure, a phenomenon previously described in patients with MS [46]. This could be attributed to reductions in neuronal communication efficiency caused by an increase in temperature [47]. In contrast, PCC patients reported that fatigue worsens with cold exposure compared to MS. Although this difference was not statistically significant after controlling for fatigue severity, this observation remains noteworthy, as it has not been previously described in the literature. This finding could be linked to dysautonomia which has been observed in PCC patients [48] and could affect temperature regulation [49], although specific studies confirming this observation and evaluating the mechanisms underlying this improvement with cold are necessary.

Moreover, patients with PCC and fibromyalgia showed no significant differences in intensity fatigue, possibly due to shared clinical and pathological mechanisms. Previous studies have shown that both conditions present a similar symptomatology such as poor sleep quality [50,51], pain, and fatigue which impact daily life activities [52]. The etiology of PCC is attributed to an infection [12]. In the same vein, the exact etiology of fibromyalgia remains unknown, but some studies suggest a potential post-viral origin [53,54]. Moreover, it has been even proposed that certain mechanisms associated with COVID-19 could trigger the onset of a fibromyalgia-like syndrome or exacerbate its symptoms [55].

Our study has some limitations. First, we invited patients to participate in the study without prior supervision of the diagnosis. However, we contacted patients’ associations and requested in our questionnaire medical confirmation of the diagnosis and medical history from participants to ensure these factors did not influence our results. Second, our sample could not be totally representative of all the PCC, fibromyalgia, and MS patients, because the present study was focused on the fatigue description in these pathologies, and, thus, we cannot evaluate the frequency of fatigue across the disorders. However, this limitation should not impact the main findings of the study, which are related to the characteristics of fatigue. Additionally, the sociodemographic characteristics of the sample are similar to those previously reported. In this regard, previous research also reported a high prevalence of fatigue symptoms in these disorders [13,15,16], specifically in patients who are typically young (range age 25 to 55) [18,56,57] and the majority female [58,59,60], which suggests that our sample may be representative of those PCC, fibromyalgia, and MS patients with fatigue. Finally, this questionnaire was developed to capture the similarities and differences among the three pathologies regarding fatigue expression. While the proposed questionnaire is broad, its design provides the necessary flexibility for future adaptations. If it were to be used for a specific clinical purpose, it would be feasible to conduct a complementary study to select the most relevant questions based on the particular clinical objective (e.g., assessing symptom intensity, analyzing specific characteristics of a particular disease, etc.). This would ensure that the instrument is optimally tailored to the needs of the application context.

## 5. Conclusions

In conclusion, our study found that fatigue is more intense in patients with PCC and fibromyalgia compared to those with MS. Additionally, differences in the characteristics of fatigue were identified: patients with MS show a greater sensitivity to heat, and mood has a greater impact. Conversely, patients with PCC and fibromyalgia have fewer benefits from rest and are more likely to develop delayed fatigue after physical or cognitive activity, which is closely associated with fatigue severity. Similarly, PCC patients reported worsening fatigue with cold exposure. Fatigue in fibromyalgia patients could be exacerbated by pain. No significant differences were observed across the three groups in terms of cognitive fatigue or difficulties in predicting the ability to perform activities. Overall, these findings suggest the existence of potential intensity and qualitative differences, which may be useful in clinical practice. Furthermore, the clinical differences suggest differences in the central and/or peripheral mechanism involved in the pathophysiology of fatigue in PCC, fibromyalgia, and MS. These findings should be considered in future interventions targeting fatigue management in these conditions.

## Figures and Tables

**Table 1:** Demographic and clinical characteristics of PCC, fibromyalgia, and MS.

		PCC	Fibromyalgia	MS	F/Chi	*p*
Demographics	Age (years)	49.81 (8.24)	52.56 (7.88)	48.15 (9.21)	7.487	0.001
	Sex, female	84.5%	95.6%	70.4%	26.103	<0.001
	Evolution (years)	3.16 (0.94)	8.67 (8.00)	11.41 (8.69)	77.630	<0.001
Clinical	MFIS alteration	99.5%	99.1%	90%	25.680	<0.001
	Hypertension	19%	16%	18.3%	1.173	0.311
	Diabetes	2.8%	6.3%	6.1%	0.807	0.447
	Dyslipidemia	22.6%	28.6%	15.3%	2.636	0.073
	Tobacco smoking	5.6%	18.8%	20.4%	9.186	<0.001
	Autoimmune disease diagnosis	20.2%	29.5%	26.5%	10.895	<0.001

Values are shown as mean (SD) or *n* (%). PCC = Post-COVID Condition; MS = multiple sclerosis; MFIS = Modified Impact Fatigue Scale.

**Table 2:** Comparison of PCC, fibromyalgia, and MS in the Intensity Scale.

Groups				ANOVA	Post Hoc Analysis		
Item	PCC	Fibromyalgia	MS	Difference Between Groups F (*p*)	PCC vs. Fibromyalgia	PCC vs. MS	Fibromyalgia vs. MS
C-1 I feel more tired	3.59 (0.75)	3.60 (0.70)	3.19 (0.96)	**9.640** **(<0.001)**	0.999	**<0.001**	**0.001**
C-2 I need to rest more hours	3.66 (0.71)	3.66 (0.62)	3.19 (0.94)	**14.390** **(<0.001)**	0.999	**<0.001**	**<0.001**
C-3 I have the sensation of lacking energy	3.79 (0.51)	3.75 (0.65)	3.31 (0.90)	**19.354** **(<0.001)**	0.888	**<0.001**	**<0.001**
C-4 I have lost interest in activities I used to do	3.05 (1.08)	3.21 (1.00)	2.66 (1.29)	**6.761** **(0.001)**	0.415	0.012	**0.001**
C-5 I feel like I have less muscle strength	3.71 (0.64)	3.63 (0.73)	3.21 (0.97)	**14.863** **(<0.001)**	0.612	**<0.001**	**<0.001**
C-6 I feel pain in my limbs	3.47 (0.99)	3.78 (0.54)	2.52 (1.40)	**43.760** **(<0.001)**	0.027	**<0.001**	**<0.001**
C-7 Pain worsens my feeling of tiredness	3.34 (0.99)	3.54 (0.79)	2.63 (1.43)	**21.411** **(<0.001)**	0.218	**<0.001**	**<0.001**
C-8 I feel like I have difficulty concentrating	3.54 (0.79)	3.61 (0.72)	2.87 (1.30)	**21.809** **(<0.001)**	0.823	**<0.001**	**<0.001**
C-9 I feel like I don’t feel like doing anything	2.93 (1.14)	3.29 (1.01)	2.73 (1.23)	**6.876** **(0.001)**	0.015	0.343	**0.001**
C-10 I have trouble starting things because I feel like I won’t be able to do them	2.81 (1.11)	3.05 (1.10)	2.58 (1.26)	4.442 (0.012)	0.158	0.237	0.009
C-11 I have trouble finishing things because I feel like I won’t be able to finish them	2.64 (1.19)	2.89 (1.14)	2.47 (1.32)	3.295 (0.038)	0.171	0.483	0.032
C-12 I feel that my tiredness is permanent	3.57 (0.71)	3.63 (0.77)	2.94 (1.25)	**20.763** **(<0.001)**	0.832	**<0.001**	**<0.001**
C-13 I feel tired regardless of the physical activity I do	3.36 (0.91)	3.53 (0.88)	2.97 (1.17)	**9.036** **(<0.001)**	0.287	0.003	**<0.001**
C-14 Tiredness prevents me from doing physical exercise/activity	3.30 (0.88)	3.21 (0.97)	2.54 (1.15)	**21.348** **(<0.001)**	0.675	**<0.001**	**<0.001**
C-15 I feel that tiredness has changed my life	3.82 (0.49)	3.65 (0.77)	3.34 (0.93)	**16.565** **(<0.001)**	0.089	**<0.001**	0.003
C-17 I notice that I am much more tired than my family or friends who also had COVID-19	3.65 (0.70)	3.78 (0.51)	3.44 (0.86)	**6.206** **(0.002)**	0.282	0.033	**0.002**
C-18 The feeling of tiredness interferes with my sexual desire	3.32 (0.98)	3.46 (0.90)	2.91 (1.24)	**8.105** **(<0.001)**	0.493	0.003	**<0.001**

Values are shown as mean (SD). Results that survived Bonferroni correction are highlighted in bold. PCC = Post-COVID Condition; MS = multiple sclerosis.

**Table 3:** Comparison of PCC, fibromyalgia, and MS in the Characteristics Scale.

Groups				ANOVA	Post Hoc Analysis		
Item	PCC	Fibromyalgia	MS	Difference Between Groups F (*p*)	PCC vs. Fibromyalgia	PCC vs. MS	Fibromyalgia vs. MS
D-1 The tiredness I have now is not the same as the tiredness I had before the COVID-19 infection or diagnosis of Fibromyalgia/Myalgic Encephalomyelitis/Chronic Fatigue Syndrome or MS	3.69 (0.90)	3.01 (1.33)	3.52 (0.75)	**16.715** **(<0.001)**	**<0.001**	0.397	**0.001**
D-2 I notice that my tiredness has significant variations in intensity throughout the day	3.03 (0.97)	2.79 (1.07)	3.19 (0.85)	4.329 (0.014)	0.090	0.395	0.012
D-3 Despite being tired, I feel like doing a task, even though I know it tires me	2.48 (1.15)	2.31 (1.24)	2.73 (0.96)	3.280 (0.039)	0.401	0.206	0.029
D-4 When it’s hot, I get tired more easily than when it’s cold	2.34 (1.37)	2.77 (1.35)	3.59 (0.81)	**30.263** **(<0.001)**	0.012	**<0.001**	**<0.001**
D-5 When it’s cold, I get tired more easily than when it’s hot	1.37 (1.20)	1.26 (1.17)	0.74 (1.03)	**9.356** **(<0.001)**	0.727	**<0.001**	0.005
D-6 When I’m in a conversation and lose track, I can follow it more easily if I take a short break	1.39 (1.18)	1.54 (1.14)	1.91 (1.26)	6.037 (0.003)	0.526	0.002	0.075
D-7 After sleeping, I wake up feeling less tired	0.98 (1.00)	0.66 (0.92)	1.80 (1.25)	**30.450** **(<0.001)**	0.025	**<0.001**	**<0.001**
D-8 I feel that tiredness worsens throughout the day if I don’t rest	3.27 (0.97)	3.15 (1.06)	3.25 (0.88)	0.489 (0.614)	0.595	0.991	0.775
D-9 I struggle to follow a movie on TV, but I follow it better if there are commercials in between	1.21 (1.16)	1.30 (1.18)	1.14 (1.12)	0.497 (0.609)	0.789	0.867	0.586
D-10. I have trouble thinking clearly when I’m busier	3.11 (1.07)	3.08 (1.05)	2.63 (1.28)	6.406 (0.002)	0.966	0.002	0.012
D-11 I can start physical exercise normally, but I have to stop sooner than usual	1.90 (1.53)	2.35 (1.35)	2.64 (1.18)	**9.506** **(<0.001)**	0.019	**<0.001**	0.348
D-12 The feeling of tiredness changes with my mood; when I’m feeling down, I feel more tired	1.33 (1.30)	2.11 (1.36)	2.76 (1.10)	**42.832** **(<0.001)**	**<0.001**	**<0.001**	**0.001**
D-13 When I wake up in the morning, I feel like I’m not tired	.65 (1.06)	.58 (1.14)	1.56 (1.42)	**22.160** **(<0.001)**	0.883	**<0.001**	**<0.001**
D-14 When I walk and have to stop due to tiredness, I recover and walk like before	.95 (1.01)	.92 (0.96)	1.59 (1.06)	**14.328** **(<0.001)**	0.962	**<0.001**	**<0.001**
D-15 When I read a book and lose focus, I recover if I take a break	1.37 (1.14)	1.22 (1.06)	2.06 (1.10)	**15.869** **(<0.001)**	0.467	**<0.001**	**<0.001**
D-16 After doing an important task, when I get up the next morning, I feel more tired than before	3.33 (0.95)	3.30 (0.91)	2.76 (1.22)	**10.872** **(<0.001)**	0.953	**<0.001**	**.001**
D-17 I feel that when I do an important activity, it consumes all my energy	3.64 (0.66)	3.65 (0.69)	3.32 (0.91)	**6.831** **(0.001)**	0.979	0.002	0.004
D-18 Sometimes, after physical activity, intense tiredness appears with a delay (for example, 12, 24, 72 h later)	2.99 (1.28)	3.09 (1.08)	2.41 (1.21)	**9.135** **(<0.001)**	0.761	**0.001**	**<0.001**
D-19 Sometimes, after cognitive activity, intense tiredness appears with a delay (for example, 12, 24, 72 h later)	2.81 (1.27)	2.75 (1.11)	2.07 (1.23)	**12.102** **(<0.001)**	0.889	**<0.001**	**<0.001**
D-20 I feel that tiredness improves after meals	0.50 (0.82)	0.69 (1.06)	0.82 (1.08)	4.065 (0.018)	0.187	0.021	0.619
D-21 I feel that tiredness worsens after meals	1.77 (1.49)	1.52 (1.45)	2.07 (1.42)	3.423 (0.034)	0.320	0.235	0.025
D-22 My tiredness is often accompanied by muscle pain	3.13 (1.10)	3.62 (0.60)	2.26 (1.41)	**39.497** **(<0.001)**	**<0.001**	**<0.001**	**<0.001**
D-23 Physical tiredness is worse than mental tiredness	2.19 (1.23)	2.07 (1.18)	2.18 (1.28)	0.366 (0.693)	0.683	0.997	0.810
D-25 The feeling of tiredness is accompanied by headaches	2.34 (1.29)	2.72 (1.11)	1.65 (1.33)	**18.018** **(<0.001)**	0.028	**<0.001**	**<0.001**
D-26 The feeling of tiredness is accompanied by joint pain	2.94 (1.23)	3.39 (0.77)	2.16 (1.39)	**27.392** **(<0.001)**	0.004	**<0.001**	**<0.001**
D-27 The feeling of tiredness disappears after resting	1.04 (0.92)	.95 (0.87)	1.74 (0.96)	**22.029** **(<0.001)**	0.699	**<0.001**	**<0.001**
D-28 I am unable to dose physical activity to predict fatigue episodes	2.60 (1.19)	2.38 (1.25)	2.39 (1.25)	1.563 (0.211)	0.293	0.364	10.000
D-29 I am unable to dose cognitive activity to predict fatigue episodes	2.68 (1.18)	2.47 (1.20)	2.40 (1.26)	2.209 (0.111)	0.296	0.148	0.902
D-30 I have “flare-ups” of worsening fatigue (flare-up: an episode of at least 24 h of significant worsening of fatigue)	3.46 (0.87)	3.36 (0.92)	2.05 (1.45)	**62.249** **(<0.001)**	0.712	**<0.001**	**<0.001**
D-31 I know what triggers these flare-ups	1.83 (1.24)	1.81 (1.27)	1.73 (1.35)	0.189 (0.827)	0.993	0.815	0.895
D-32 Activities that motivate me generate less fatigue	0.74 (0.94)	1.39 (1.07)	1.51 (1.20)	**23.862** **(<0.001)**	**<0.001**	**<0.001**	0.698

Values are shown as mean (SD). Results that survived Bonferroni correction are highlighted in bold.. PCC = Post-COVID Condition; MS = multiple sclerosis.

**Table 4:** Characteristics Scale adjusted for covariates including age and fatigue severity (MFIS).

	COVARIATE AGE				COVARIATE MFIS			
	ANCOVA	Post Hoc Analysis			ANCOVA	Post Hoc Analysis		
Item	Difference Between Groups F (*p*)	PCC vs. Fibromyalgia	PCC vs. MS	Fibromyalgia vs. MS	Difference Between Groups F (*p*)	PCC vs. Fibromyalgia	PCC vs. MS	Fibromyalgia vs. MS
D-1 The tiredness I have now is not the same as the tiredness I had before the COVID-19 infection or diagnosis of Fibromyalgia/Myalgic Encephalomyelitis/Chronic Fatigue Syndrome or MS	**15.758** **(<0.001)**	**<0.001**	0.406	0.002	**16.716** **(<0.001)**	**<0.001**	0.777	**<0.001**
D-2 I notice that my tiredness has significant variations in intensity throughout the day	4.134 (0.017)	0.096	0.402	0.014	4.457 (0.012)	0.097	0.343	0.011
D-3 Despite being tired, I feel like doing a task, even though I know it tires me	3.883 (0.021)	0.299	0.165	0.015	1.733 (0.178)	0.294	0.827	0.198
D-4 When it’s hot, I get tired more easily than when it’s cold	**26.671** **(<0.001)**	0.009	**<0.001**	**<0.001**	**23.326** **(<0.001)**	0.016	**<0.001**	**<0.001**
D-5 When it’s cold, I get tired more easily than when it’s hot	**9.048** **(<0.001)**	0.625	**<0.001**	0.010	5.967 (0.003)	0.797	0.002	0.028
D-6 When I’m in a conversation and lose track, I can follow it more easily if I take a short break	6.069 (0.003)	0.449	0.002	0.103	3.092 (0.046)	0.618	0.036	0.296
D-7 After sleeping, I wake up feeling less tired	**29.915** **(<0.001)**	0.031	**<0.001**	**<0.001**	**22.685** **(<0.001)**	0.014	**<0.001**	**<0.001**
D-8 I feel that tiredness worsens throughout the day if I don’t rest	0.733 (0.481)	0.471	0.998	0.638	0.973 (0.379)	0.696	0.688	0.347
D-9 I struggle to follow a movie on TV, but I follow it better if there are commercials in between	0.521 (0.595)	0.750	0.883	0.577	0.579 (0.561)	0.636	0.682	10.000
D-10. I have trouble thinking clearly when I’m busier	5.908 (0.003)	0.900	0.002	0.027	0.093 (0.911)	0.919	0.998	0.931
D-11 I can start physical exercise normally, but I have to stop sooner than usual	**9.512** **(<0.001)**	0.071	**<0.001**	0.140	**8.230** **(<0.001)**	0.021	**<0.001**	0.438
D-12 The feeling of tiredness changes with my mood; when I’m feeling down, I feel more tired	**42.997** **(<0.001)**	**<0.001**	**<0.001**	**<0.001**	**42.068** **(<0.001)**	**<0.001**	**<0.001**	**<0.001**
D-13 When I wake up in the morning, I feel like I’m not tired	**23.906** **(<0.001)**	0.767	**<0.001**	**<0.001**	**16.006** **(<0.001)**	0.813	**<0.001**	**<0.001**
D-14 When I walk and have to stop due to tiredness, I recover and walk like before	**14.174** **(<0.001)**	0.973	**<0.001**	**<0.001**	**8.187** **(<0.001)**	0.881	**<0.001**	**<0.001**
D-15 When I read a book and lose focus, I recover if I take a break	**16.039** **(<0.001)**	0.541	**<0.001**	**<0.001**	**10.926** **(<0.001)**	0.376	**<0.001**	**<0.001**
D-16 After doing an important task, when I get up the next morning, I feel more tired than before	**10.100** **(<0.001)**	0.890	**<0.001**	**<0.001**	4.487 (0.012)	10.000	0.013	0.029
D-17 I feel that when I do an important activity, it consumes all my energy	5.939 (0.003)	10.000	0.003	0.012	0.992 (0.372)	0.776	0.603	0.338
D-18 Sometimes, after physical activity, intense tiredness appears with a delay (for example, 12, 24, 72 h later)	**9.158** **(** **<0.001** **)**	0.703	**<0.001**	**<0.001**	4.933 (0.008)	0.636	0.028	0.007
D-19 Sometimes, after cognitive activity, intense tiredness appears with a delay (for example, 12, 24, 72 h later)	**11.203** **(** **<0.001** **)**	0.857	**<0.001**	**<0.001**	4.271 (0.015)	0.991	0.014	0.041
D-20 I feel that tiredness improves after meals	4.372 (0.013)	0.192	0.015	0.563	2.755 (0.065)	0.218	0.094	0.846
D-21 I feel that tiredness worsens after meals	2.560 (0.079)	0.420	0.328	0.062	4.121 (0.017)	0.359	0.120	0.012
D-22 My tiredness is often accompanied by muscle pain	**39.985** **(** **<0.001** **)**	<00.001	**<0.001**	**<0.001**	**27.995** **(<0.001)**	**<0.001**	**<0.001**	**<0.001**
D-23 Physical tiredness is worse than mental tiredness	0.192 (0.825)	0.813	0.995	0.911	0.621 (0.538)	0.595	0.690	0.999
D-25 The feeling of tiredness is accompanied by headaches	**16.926** **(** **<0.001** **)**	0.036	**<0.001**	**<0.001**	**10.490** **(<0.001)**	0.006	0.070	**<0.001**
D-26 The feeling of tiredness is accompanied by joint pain	**26.015** **(** **<0.001** **)**	0.004	**<0.001**	**<0.001**	**19.160** **(<0.001)**	**<0.001**	**<0.001**	**<0.001**
D-27 The feeling of tiredness disappears after resting	**23.602** **(** **<0.001** **)**	0.563	**<0.001**	**<0.001**	**13.912** **(<0.001)**	0.548	**<0.001**	**<0.001**
D-28 I am unable to dose physical activity to predict fatigue episodes	1.748 (0.175)	0.250	0.339	0.997	1.320 (0.268)	0.446	0.794	0.268
D-29 I am unable to dose cognitive activity to predict fatigue episodes	2.418 (0.099)	0.260	0.147	0.931	0.965 (0.382)	0.462	0.946	0.435
D-30 I have “flare-ups” of worsening fatigue (flare-up: an episode of at least 24 h of significant worsening of fatigue)	**63.262** **(** **<0.001** **)**	0.867	**<0.001**	**<0.001**	**41.257** **(<0.001)**	0.898	**<0.001**	**<0.001**
D-31 I know what triggers these flare-ups	0.237 (0.789)	0.998	0.800	0.818	0.088 (0.915)	0.996	0.909	0.948
D-32 Activities that motivate me generate less fatigue	**23.453** **(** **<0.001** **)**	**<0.001**	**<0.001**	0.504	**19.663** **(<0.001)**	**<0.001**	**<0.001**	0.955

Results that survived Bonferroni correction are highlighted in bold. MFIS = Modified Impact Fatigue Scale.

## Data Availability

The datasets used and/or analyzed during the current study are available from the corresponding author upon reasonable request, due to ethical committee restrictions.

## Supplementary Materials

The following supporting information can be downloaded at: https://www.mdpi.com/article/10.3390/jcm14030952/s1, Fatigue Intensity and Characteristics Scale (FICS) and Supplementary Table S1: Intensity Scale, adjusted for age as a covariate.
