# Supplementary material for: Differential Fatigue Profile in Patients with Post-COVID Condition, Fibromyalgia, and Multiple Sclerosis
Source: J Clin Med. 2025 Feb 2;14(3):952. doi: 10.3390/jcm14030952 (PMC11818582; doi:10.3390/jcm14030952)
Supplement: Supplementary file 1 [file jcm-14-00952-s001.zip › jcm-3447235-supplementary.pdf]

Supplementary Materials

Differential Fatigue Profile in Patients with Post-COVID Condition, Fibromyalgia, and Multiple Sclerosis

Authors: Silvia Oliver-Mas, Jordi A Matias-Guiu, Cristina Delgado-Alonso, Constanza Cuevas, José Manuel Alcalá Ramírez del Puerto, Juan Ignacio López-Carbonero, Jorge Matias-Guiu, Maria Diez-Cirarda

Affiliation: Department of Neurology. Hospital Clínico San Carlos. San Carlos Health Research Institute (IdISSC), Universidad Complutense de Madrid.

Supplementary Files:

1) **Fatigue Intensity and Characteristics Scale (FICS)**
2) **Supplementary Table S1**

Fatigue Intensity and Characteristics Scale (FICS)

Scale A. Questionnaire of demographic and clinical characteristics of the participants

Clinical and demographic characteristics	
Please answer 'Yes', 'No', or 'Don't know' to the questions, unless a specific answer is requested	
Items	Options/response
A-1 Gender	o Male<br>o Female<br>o No binary or others
A-2 Age (in years)	Open response
A-3 Have you been diagnosed with high blood pressure by your doctor?	o Yes, before the COVID-19 infection or diagnosis of Fibromyalgia/Myalgic Encephalomyelitis/Chronic Fatigue Syndrome or MS<br>o Yes, after the COVID-19 infection or diagnosis of Fibromyalgia/Myalgic Encephalomyelitis/Chronic Fatigue Syndrome or MS<br>o No<br>o I don't know
A-4 Have you been diagnosed with diabetes mellitus by your doctor?	o Yes, before the COVID-19 infection or diagnosis of Fibromyalgia/Myalgic Encephalomyelitis/Chronic Fatigue Syndrome or MS<br>o Yes, after the COVID-19 infection or diagnosis of Fibromyalgia/Myalgic Encephalomyelitis/Chronic Fatigue Syndrome or MS<br>o No<br>o I don't know
A-5 Are you taking treatment to lower cholesterol or other fats in the blood?	o Yes, since before the COVID-19 infection or diagnosis of Fibromyalgia/Myalgic Encephalomyelitis/Chronic Fatigue Syndrome or MS<br>o Yes, after the COVID-19 infection or diagnosis of Fibromyalgia/Myalgic Encephalomyelitis/Chronic Fatigue Syndrome or MS<br>o No<br>o I don't know
A-6 Do you consume alcohol daily?	o Yes, since before the COVID-19 infection or diagnosis of Fibromyalgia/Myalgic Encephalomyelitis/Chronic Fatigue Syndrome or MS<br>o Yes, after the COVID-19 infection or diagnosis of Fibromyalgia/Myalgic Encephalomyelitis/Chronic Fatigue Syndrome or MS<br>o No<br>o I don't know
A-7 Do you consume any drugs or psychoactive substances daily?	o Yes, since before the COVID-19 infection or diagnosis of Fibromyalgia/Myalgic Encephalomyelitis/Chronic Fatigue Syndrome or MS

	○ Yes, after the COVID-19 infection or diagnosis of Fibromyalgia/Myalgic Encephalomyelitis/Chronic Fatigue Syndrome or MS<br>○ No<br>○ I don't know
A-8 Do you consume tobacco daily?	○ Yes, since before the COVID-19 infection or diagnosis of Fibromyalgia/Myalgic Encephalomyelitis/Chronic Fatigue Syndrome or MS<br>○ Yes, after the COVID-19 infection or diagnosis of Fibromyalgia/Myalgic Encephalomyelitis/Chronic Fatigue Syndrome or MS<br>○ No<br>○ I don't know
A-9 Are you diagnosed by your doctor with chronic fatigue syndrome or fibromyalgia? *(This question is just in PCC and MS questionnaire)	○ Yes, before the COVID-19 infection or diagnosis of Fibromyalgia/Myalgic Encephalomyelitis/Chronic Fatigue Syndrome or MS<br>○ Yes, after the COVID-19 infection or diagnosis of Fibromyalgia/Myalgic Encephalomyelitis/Chronic Fatigue Syndrome or MS<br>○ No<br>○ I don't know
A-10 Are you diagnosed by your doctor with any autoimmune disease (rheumatoid arthritis, hyperthyroidism, ankylosing spondylitis, etc.)?	○ Yes, before the COVID-19 infection or diagnosis of Fibromyalgia/Myalgic Encephalomyelitis/Chronic Fatigue Syndrome or MS<br>○ Yes, after the COVID-19 infection or diagnosis of Fibromyalgia/Myalgic Encephalomyelitis/Chronic Fatigue Syndrome or MS<br>○ No<br>○ I don't know
A-11 Are you diagnosed by your doctor with postural tachycardia syndrome (POTS)?	○ Yes, before the COVID-19 infection or diagnosis of Fibromyalgia/Myalgic Encephalomyelitis/Chronic Fatigue Syndrome or MS<br>○ Yes, after the COVID-19 infection or diagnosis of Fibromyalgia/Myalgic Encephalomyelitis/Chronic Fatigue Syndrome or MS<br>○ No<br>○ I don't know
A-12 Do you have, or have you had a cancer diagnosis?	○ Yes, before the COVID-19 infection or diagnosis of Fibromyalgia/Myalgic Encephalomyelitis/Chronic Fatigue Syndrome or MS<br>○ Yes, after the COVID-19 infection or diagnosis of Fibromyalgia/Myalgic Encephalomyelitis/Chronic Fatigue Syndrome or MS<br>○ No<br>○ I don't know
A-13 Are you diagnosed by your doctor with any neuromuscular disease (peripheral	○ Yes, before the COVID-19 infection or diagnosis of Fibromyalgia/Myalgic

neuropathy, myopathy, myasthenia gravis, etc.)?	Encephalomyelitis/Chronic Fatigue Syndrome or MS<br>○ Yes, after the COVID-19 infection or diagnosis of Fibromyalgia/Myalgic Encephalomyelitis/Chronic Fatigue Syndrome or MS<br>○ No<br>○ I don't know
A-14 Are you diagnosed by your doctor with chronic kidney disease?	○ Yes, before the COVID-19 infection or diagnosis of Fibromyalgia/Myalgic Encephalomyelitis/Chronic Fatigue Syndrome or MS<br>○ Yes, after the COVID-19 infection or diagnosis of Fibromyalgia/Myalgic Encephalomyelitis/Chronic Fatigue Syndrome or MS<br>○ No<br>○ I don't know
A-15 Are you diagnosed by your doctor with chronic obstructive pulmonary disease (COPD)?	○ Yes, before the COVID-19 infection or diagnosis of Fibromyalgia/Myalgic Encephalomyelitis/Chronic Fatigue Syndrome or MS<br>○ Yes, after the COVID-19 infection or diagnosis of Fibromyalgia/Myalgic Encephalomyelitis/Chronic Fatigue Syndrome or MS<br>○ No<br>○ I don't know
A-16 Are you diagnosed by your doctor with asthma?	○ Yes, before the COVID-19 infection or diagnosis of Fibromyalgia/Myalgic Encephalomyelitis/Chronic Fatigue Syndrome or MS<br>○ Yes, after the COVID-19 infection or diagnosis of Fibromyalgia/Myalgic Encephalomyelitis/Chronic Fatigue Syndrome or MS<br>○ No<br>○ I don't know
A-17 Are you diagnosed by your doctor with sleep disorders (sleep apnea, narcolepsy, intermittent insomnia, etc.)?	○ Yes, before the COVID-19 infection or diagnosis of Fibromyalgia/Myalgic Encephalomyelitis/Chronic Fatigue Syndrome or MS<br>○ Yes, after the COVID-19 infection or diagnosis of Fibromyalgia/Myalgic Encephalomyelitis/Chronic Fatigue Syndrome or MS<br>○ No<br>○ I don't know
A-18 Are you diagnosed by your doctor with heart problems (tachycardia, bradycardia, myocardial infarction, etc.)?	○ Yes, before the COVID-19 infection or diagnosis of Fibromyalgia/Myalgic Encephalomyelitis/Chronic Fatigue Syndrome or MS<br>○ Yes, after the COVID-19 infection or diagnosis of Fibromyalgia/Myalgic Encephalomyelitis/Chronic Fatigue Syndrome or MS<br>○ No<br>○ I don't know
A-19 Do you regularly take antidepressants for the treatment of depression?	○ Yes, since before the COVID-19 infection or diagnosis of

	Fibromyalgia/Myalgic Encephalomyelitis/Chronic Fatigue Syndrome or MS<br>○ Yes, after the COVID-19 infection or diagnosis of Fibromyalgia/Myalgic Encephalomyelitis/Chronic Fatigue Syndrome or MS<br>○ No<br>○ I don't know
A-20 Do you regularly take antihistamines for the treatment of allergies or other disorders?	○ Yes, since before the COVID-19 infection or diagnosis of Fibromyalgia/Myalgic Encephalomyelitis/Chronic Fatigue Syndrome or MS<br>○ Yes, after the COVID-19 infection or diagnosis of Fibromyalgia/Myalgic Encephalomyelitis/Chronic Fatigue Syndrome or MS<br>○ No<br>○ I don't know
A-21 Do you regularly take omeprazole or similar medications for digestive problems or other disorders?	○ Yes, since before the COVID-19 infection or diagnosis of Fibromyalgia/Myalgic Encephalomyelitis/Chronic Fatigue Syndrome or MS<br>○ Yes, after the COVID-19 infection or diagnosis of Fibromyalgia/Myalgic Encephalomyelitis/Chronic Fatigue Syndrome or MS<br>○ No<br>○ I don't know
A-22 Do you take benzodiazepine sedatives (e.g., Orfidal or similar) daily for sleep problems?	○ Yes, since before the COVID-19 infection or diagnosis of Fibromyalgia/Myalgic Encephalomyelitis/Chronic Fatigue Syndrome or MS<br>○ Yes, after the COVID-19 infection or diagnosis of Fibromyalgia/Myalgic Encephalomyelitis/Chronic Fatigue Syndrome or MS<br>○ No<br>○ I don't know
A-23 Do you regularly take neuroleptics or antipsychotics as prescribed by the mental health centre?	○ Yes, since before the COVID-19 infection or diagnosis of Fibromyalgia/Myalgic Encephalomyelitis/Chronic Fatigue Syndrome or MS<br>○ Yes, after the COVID-19 infection or diagnosis of Fibromyalgia/Myalgic Encephalomyelitis/Chronic Fatigue Syndrome or MS<br>○ No<br>○ I don't know
A-24 Do you regularly take diuretics as prescribed by your doctor for the treatment of high blood pressure?	○ Yes, since before the COVID-19 infection or diagnosis of Fibromyalgia/Myalgic Encephalomyelitis/Chronic Fatigue Syndrome or MS<br>○ Yes, after the COVID-19 infection or diagnosis of Fibromyalgia/Myalgic

	Encephalomyelitis/Chronic Fatigue Syndrome or MS<br>○ No<br>○ I don't know
A-25 Have you been diagnosed by your doctor with any of these diseases before the diagnosis of Fibromyalgia or Myalgic Encephalomyelitis/Chronic Fatigue Syndrome? If you select the option 'Other autoimmune disease' (please specify which one in the next question, A-25)	○ Addison's disease<br>○ Celiac disease<br>○ Thyroid disease<br>○ Rheumatoid arthritis<br>○ Sjögren's syndrome<br>○ Ulcerative colitis / Crohn's disease<br>○ Other autoimmune disease<br>○ I have not been diagnosed with any of the previous diseases
A-26 If you selected 'Other autoimmune disease' in the previous question, please specify which one.	Open response
A-27 Have you been diagnosed by your doctor with any of these diseases after the diagnosis of Fibromyalgia or Myalgic Encephalomyelitis/Chronic Fatigue Syndrome? If you select the option 'Other autoimmune disease' (please specify which one in the next question, A-27)	○ Addison's disease<br>○ Celiac disease<br>○ Thyroid disease<br>○ Rheumatoid arthritis<br>○ Sjögren's syndrome<br>○ Ulcerative colitis / Crohn's disease<br>○ Other autoimmune disease<br>○ I have not been diagnosed with any of the previous diseases
A-28 If you selected 'Other autoimmune disease' in the previous question, please specify which one	Open response
A-29 Please indicate any other medical history (before the diagnosis of Fibromyalgia or Myalgic Encephalomyelitis/Chronic Fatigue Syndrome) that you consider relevant, or any disease not mentioned in the questionnaire. If you do not wish to mention any other medical diagnosis, please respond 'no'.	Open response

Scale B. Questionnaire about COVID-19 infections and vaccination

Questionnaire about COVID-19 infections and vaccination	
Please answer 'Yes', 'No', or 'Don't know' to the questions, unless a specific answer is requested	
Items	Options/response
B-1 Have you been infected with COVID-19? If not, please go directly to question B-53 *This question is only in the fibromyalgia and MS questionnaire	○ Yes<br>○ No
B-2 Date or the approximate date when you first had COVID-19 (e.g., January 7, 2019)	Open response
B-3 Was your first episode of COVID-19 symptomatic or asymptomatic? (Asymptomatic means complete absence of symptoms).	○ Symptomatic<br>○ Asymptomatic (without symptoms)<br>○ I don't know
B-4 Did your first episode of COVID-19 require you to go to the emergency room?	○ Yes<br>○ No<br>○ I don't know
B-5 Did your first episode of COVID-19 require you to go to intensive care?	○ Yes<br>○ No<br>○ I don't know
B-6 Did your first episode of COVID-19 require you to use assisted ventilation?	○ Yes<br>○ No<br>○ I don't know
B-7 If you needed assisted ventilation, how many days did you need it? (If you did not need assisted ventilation, please answer 0)	Open response
B-8 Were you hospitalized for your first episode of COVID-19?	○ Yes<br>○ No<br>○ I don't know
B-9 If you were hospitalized, how many days were you hospitalized?	Open response
B-10 In your first episode of COVID-19, did you experience olfactory changes (loss or alteration of taste or smell)?	○ Yes<br>○ No<br>○ I don't know
B-11 Did the olfactory or taste changes last more than three months?	○ Yes<br>○ No<br>○ I don't know
B-12 In your first episode of COVID-19, did you experience severe headache (head pain)?	○ Yes<br>○ No<br>○ I don't know
B-13 In your first episode of COVID-19, did you experience a headache for more than three months?	○ Yes<br>○ No<br>○ I don't know
B-14 In your first episode of COVID-19, did you experience any episodes of confusion?	○ Yes<br>○ No<br>○ I don't know
B-15 In your first episode of COVID-19, did you experience memory or concentration issues during the acute phase?	○ Yes<br>○ No<br>○ I don't know
B-16 Did the memory or concentration issues last more than 3 months?	○ Yes<br>○ No

	○ I don't know
B-17 In your first episode of COVID-19, did you experience depression during the acute phase?	○ Yes<br>○ No<br>○ I don't know
B-18 Did the depressive symptoms last more than three months?	○ Yes<br>○ No<br>○ I don't know
B-19 Have you had a second episode of COVID-19? If you have not had any more episodes of COVID-19, you may go directly to question B-53.	○ Yes<br>○ No<br>○ I don't know
B-20 Date or approximate date of second COVID-19 infection (e.g., January 7, 2019)	Open response
B-21 Was your second episode of COVID-19 symptomatic or asymptomatic? (Asymptomatic means complete absence of symptoms).	○ Symptomatic<br>○ Asymptomatic (without symptoms)<br>○ I don't know
B-22 Did your second episode of COVID-19 require you to be hospitalized?	○ Yes<br>○ No<br>○ I don't know
B-23 Did your second episode of COVID-19 require you to be admitted to intensive care?	○ Yes<br>○ No<br>○ I don't know
B-24 Did your second episode of COVID-19 require you to use assisted ventilation?	○ Yes<br>○ No<br>○ I don't know
B-25 Days of hospitalization for your second episode of COVID-19 (if you were not hospitalized, please answer 0)	Open response
B-26 In your second episode of COVID-19, did you experience olfactory changes (loss or alteration of taste or smell)?	○ Yes<br>○ No<br>○ I don't know
B-27 Did the olfactory or taste changes last more than three months?	○ Yes<br>○ No<br>○ I don't know
B-28 In your second episode of COVID-19, did you experience severe headache (head pain)?	○ Yes<br>○ No<br>○ I don't know
B-29 In your second episode of COVID-19, did the headache last more than three months?	○ Yes<br>○ No<br>○ I don't know
B-30 In your second episode of COVID-19, did you experience any episodes of confusion?	○ Yes<br>○ No<br>○ I don't know
B-31 In your second episode of COVID-19, did you experience memory or concentration issues?	○ Yes<br>○ No<br>○ I don't know
B-32 Did the memory or concentration issues last more than three months?	○ Yes

	○ No<br>○ I don't know
B-33 In your second episode of COVID-19, did you experience depression during the acute phase?	○ Yes<br>○ No<br>○ I don't know
B-34 Did the depressive symptoms last more than three months?	○ Yes<br>○ No<br>○ I don't know
B-35 Have you had a third episode of COVID-19? If you have not had any more episodes of COVID-19, you may go directly to question B-53	○ Yes<br>○ No<br>○ I don't know
B-36 Date or approximate date when you had COVID-19 for the third time (e.g., January 7, 2019)	Open response
B-37 Was your third episode of COVID-19 symptomatic or asymptomatic? (Asymptomatic means complete absence of symptoms)	○ Symptomatic<br>○ Asymptomatic (without symptoms)<br>○ I don't know
B-38 Did your third episode of COVID-19 require you to be hospitalized?	○ Yes<br>○ No<br>○ I don't know
B-39 Did your third episode of COVID-19 require you to be admitted to intensive care?	○ Yes<br>○ No<br>○ I don't know
B-40 Did your third episode of COVID-19 require you to use assisted ventilation?	○ Yes<br>○ No<br>○ I don't know
B-41 Days of hospitalization for your third episode of COVID-19	Open response
B-42 In your third episode of COVID-19, did you experience olfactory changes? (loss or alteration of taste or smell).	○ Yes<br>○ No<br>○ I don't know
B-43 Did the olfactory or taste changes last more than three months?	○ Yes<br>○ No<br>○ I don't know
B-44 In your third episode of COVID-19, did you experience severe headache (head pain)?	○ Yes<br>○ No<br>○ I don't know
B-45 In your third episode of COVID-19, did the headache last more than three months?	○ Yes<br>○ No<br>○ I don't know
B-46 In your third episode of COVID-19, did you experience any episodes of confusion?	○ Yes<br>○ No<br>○ I don't know
B-47 In your third episode of COVID-19, did you experience memory or concentration issues?	○ Yes<br>○ No<br>○ I don't know

B-48 Did the memory or concentration issues last more than 3 months?	○ Yes<br>○ No<br>○ I don't know
B-49 In your third episode of COVID-19, did you experience depression during the acute phase?	○ Yes<br>○ No<br>○ I don't know
B-50 Did the depressive symptoms last more than three months?	○ Yes<br>○ No<br>○ I don't know
B-51 In addition to the previously mentioned episodes, have you had another episode of COVID-19? If yes, please specify:<br>• Date or approximate date (please answer in the following format: mm/yyyy).<br>• Description of the acute phase (hospitalization, admission to intensive care, need for assisted mechanical ventilation).<br>• Symptomatic or asymptomatic. If symptomatic, please consider whether it included any of the symptoms mentioned earlier (headaches, fatigue, memory or concentration issues, and depressive mood)."	Open response
B-52 In any of the COVID-19 episodes, has the fatigue you previously experienced due to fibromyalgia or myalgic encephalomyelitis/chronic fatigue syndrome been significantly and persistently altered? If the fatigue has not changed after the COVID-19 episode(s), please answer 'no'.	Open response
B-53 Have you received the COVID-19 vaccine?	○ Yes<br>○ No
B-54 If you have received the vaccine, was it before or after the COVID-19 infection?	○ I have received the vaccine before the COVID-19 infection<br>○ I have received the vaccine after the COVID-19 infection<br>○ I have not received any dose of the COVID-19 vaccine
B-55 In case you have received the vaccine, how many doses have you received?	○ 1 dose<br>○ 2 dose<br>○ 3 dose<br>○ 4 dose<br>○ I have not received any dose of the COVID-19 vaccine
B-56 First vaccine	○ Pfizer<br>○ AstraZeneca<br>○ Janssen<br>○ Moderna<br>○ Other<br>○ I don’t know
B-57 Second vaccine.	○ Pfizer<br>○ AstraZeneca<br>○ Janssen<br>○ Moderna

	○ Other<br>○ I don't know
B-58 Third vaccine	○ Pfizer<br>○ AstraZeneca<br>○ Janssen<br>○ Moderna<br>○ Other<br>○ I don't know
B-59 Fourth vaccine	○ Pfizer<br>○ AstraZeneca<br>○ Janssen<br>○ Moderna<br>○ Other<br>○ I don't know
B-60 In case you have received the vaccine, has your fatigue changed significantly and persistently after vaccination?	○ My fatigue improved significantly after the vaccination<br>○ My fatigue improved a little after the vaccination<br>○ I did not experience any improvement or worsening of my fatigue after the vaccination<br>○ My fatigue worsened a little after the vaccination<br>○ My fatigue worsened significantly after the vaccination
B-61 If you experienced an improvement after vaccination, how long did that improvement last?	Open response

Scale C. Intensity Scale

Intensity Scale	
In this section, we will try to describe the characteristics of the fatigue you may have experienced since being diagnosed with Fibromyalgia or Myalgic Encephalomyelitis/Chronic Fatigue Syndrome. Please consider the last 3 months when answering.<br>Please rate on a scale from 1 to 5 according to your level of agreement with the statement, where: 1. Strongly disagree; 2. Slightly agree; 3. Moderately agree; 4. Quite agree; 5. Strongly agree.	
Items	Options/response
C-1 I feel more tired	○ Strongly disagree<br>○ Slightly agree<br>○ Moderately agree<br>○ Quite agree<br>○ Strongly agree
C-2 I need to rest more hours	○ Strongly disagree<br>○ Slightly agree<br>○ Moderately agree<br>○ Quite agree<br>○ Strongly agree
C-3 I have the sensation of lacking energy	○ Strongly disagree<br>○ Slightly agree<br>○ Moderately agree<br>○ Quite agree<br>○ Strongly agree
C-4 I have lost interest in activities I used to do	○ Strongly disagree<br>○ Slightly agree<br>○ Moderately agree<br>○ Quite agree<br>○ Strongly agree
C-5 I feel like I have less muscle strength	○ Strongly disagree<br>○ Slightly agree<br>○ Moderately agree<br>○ Quite agree<br>○ Strongly agree
C-6 I feel pain in my limbs	○ Strongly disagree<br>○ Slightly agree<br>○ Moderately agree<br>○ Quite agree<br>○ Strongly agree
C-7 Pain worsens my feeling of tiredness	○ Strongly disagree<br>○ Slightly agree<br>○ Moderately agree<br>○ Quite agree<br>○ Strongly agree
C-8 I feel like I have difficulty concentrating	○ Strongly disagree<br>○ Slightly agree<br>○ Moderately agree<br>○ Quite agree<br>○ Strongly agree
C-9 I feel like I don’t feel like doing anything	○ Strongly disagree<br>○ Slightly agree<br>○ Moderately agree<br>○ Quite agree<br>○ Strongly agree
C-10 I have trouble starting things because I feel like I won’t be able to do them	○ Strongly disagree<br>○ Slightly agree<br>○ Moderately agree<br>○ Quite agree<br>○ Strongly agree

C-11 I have trouble finishing things because I feel like I won’t be able to finish them	○ Strongly disagree<br>○ Slightly agree<br>○ Moderately agree<br>○ Quite agree<br>○ Strongly agree
C-12 I feel that my tiredness is permanent	○ Strongly disagree<br>○ Slightly agree<br>○ Moderately agree<br>○ Quite agree<br>○ Strongly agree
C-13 I feel tired regardless of the physical activity I do	○ Strongly disagree<br>○ Slightly agree<br>○ Moderately agree<br>○ Quite agree<br>○ Strongly agree
C-14 Tiredness prevents me from doing physical exercise/activity	○ Strongly disagree<br>○ Slightly agree<br>○ Moderately agree<br>○ Quite agree<br>○ Strongly agree
C-15 I feel that tiredness has changed my life	○ Strongly disagree<br>○ Slightly agree<br>○ Moderately agree<br>○ Quite agree<br>○ Strongly agree
C-16 Even though I feel low-spirited, I don’t feel that tiredness is due to this; instead, I feel bad because I am tired	○ Strongly disagree<br>○ Slightly agree<br>○ Moderately agree<br>○ Quite agree<br>○ Strongly agree
C-17 I notice that I am much more tired than my family or friends who also had COVID-19	○ Strongly disagree<br>○ Slightly agree<br>○ Moderately agree<br>○ Quite agree<br>○ Strongly agree
C-18 The feeling of tiredness interferes with my sexual desire	○ Strongly disagree<br>○ Slightly agree<br>○ Moderately agree<br>○ Quite agree<br>○ Strongly agree

Scale D. Characteristics Scale

Characteristics Scale	
Please rate on the scale from 1 to 5 according to your level of agreement with the statement, where: 1. Strongly disagree; 2. Slightly agree; 3. Moderately agree; 4. Quite agree; 5. Strongly agree.	
Items	Options/response
D-1 The tiredness I have now is not the same as the tiredness I had before the COVID-19 infection or diagnosis of Fibromyalgia/Myalgic Encephalomyelitis/Chronic Fatigue Syndrome or MS	○ Strongly disagree<br>○ Slightly agree<br>○ Moderately agree<br>○ Quite agree<br>○ Strongly agree
D-2 I notice that my tiredness has significant variations in intensity throughout the day	○ Strongly disagree<br>○ Slightly agree<br>○ Moderately agree<br>○ Quite agree<br>○ Strongly agree
D-3 Despite being tired, I feel like doing a task, even though I know it tires me	○ Strongly disagree<br>○ Slightly agree<br>○ Moderately agree<br>○ Quite agree<br>○ Strongly agree
D-4 When it's hot, I get tired more easily than when it's cold	○ Strongly disagree<br>○ Slightly agree<br>○ Moderately agree<br>○ Quite agree<br>○ Strongly agree
D-5 When it's cold, I get tired more easily than when it's hot	○ Strongly disagree<br>○ Slightly agree<br>○ Moderately agree<br>○ Quite agree<br>○ Strongly agree
D-6 When I'm in a conversation and lose track, I can follow it more easily if I take a short break	○ Strongly disagree<br>○ Slightly agree<br>○ Moderately agree<br>○ Quite agree<br>○ Strongly agree
D-7 After sleeping, I wake up feeling less tired	○ Strongly disagree<br>○ Slightly agree<br>○ Moderately agree<br>○ Quite agree<br>○ Strongly agree
D-8 I feel that tiredness worsens throughout the day if I don't rest	○ Strongly disagree<br>○ Slightly agree<br>○ Moderately agree<br>○ Quite agree<br>○ Strongly agree
D-9 I struggle to follow a movie on TV, but I follow it better if there are commercials in between	○ Strongly disagree<br>○ Slightly agree<br>○ Moderately agree<br>○ Quite agree<br>○ Strongly agree
D-10. I have trouble thinking clearly when I'm busier	○ Strongly disagree<br>○ Slightly agree<br>○ Moderately agree<br>○ Quite agree<br>○ Strongly agree

D-11 I can start physical exercise normally, but I have to stop sooner than usual	○ Strongly disagree<br>○ Slightly agree<br>○ Moderately agree<br>○ Quite agree<br>○ Strongly agree
D-12 The feeling of tiredness changes with my mood; when I'm feeling down, I feel more tired	○ Strongly disagree<br>○ Slightly agree<br>○ Moderately agree<br>○ Quite agree<br>○ Strongly agree
D-13 When I wake up in the morning, I feel like I'm not tired	○ Strongly disagree<br>○ Slightly agree<br>○ Moderately agree<br>○ Quite agree<br>○ Strongly agree
D-14 When I walk and have to stop due to tiredness, I recover and walk like before	○ Strongly disagree<br>○ Slightly agree<br>○ Moderately agree<br>○ Quite agree<br>○ Strongly agree
D-15 When I read a book and lose focus, I recover if I take a break	○ Strongly disagree<br>○ Slightly agree<br>○ Moderately agree<br>○ Quite agree<br>○ Strongly agree
D-16 After doing an important task, when I get up the next morning, I feel more tired than before	○ Strongly disagree<br>○ Slightly agree<br>○ Moderately agree<br>○ Quite agree<br>○ Strongly agree
D-17 I feel that when I do an important activity, it consumes all my energy	○ Strongly disagree<br>○ Slightly agree<br>○ Moderately agree<br>○ Quite agree<br>○ Strongly agree
D-18 Sometimes, after physical activity, intense tiredness appears with a delay (for example, 12, 24, 72 hours later)	○ Strongly disagree<br>○ Slightly agree<br>○ Moderately agree<br>○ Quite agree<br>○ Strongly agree
D-19 Sometimes, after cognitive activity, intense tiredness appears with a delay (for example, 12, 24, 72 hours later)	○ Strongly disagree<br>○ Slightly agree<br>○ Moderately agree<br>○ Quite agree<br>○ Strongly agree
D-20 I feel that tiredness improves after meals	○ Strongly disagree<br>○ Slightly agree<br>○ Moderately agree<br>○ Quite agree<br>○ Strongly agree
D-21 I feel that tiredness worsens after meals	○ Strongly disagree<br>○ Slightly agree<br>○ Moderately agree<br>○ Quite agree<br>○ Strongly agree
D-22 My tiredness is often accompanied by muscle pain	○ Strongly disagree<br>○ Slightly agree<br>○ Moderately agree<br>○ Quite agree<br>○ Strongly agree

D-23 Physical tiredness is worse than mental tiredness	○ Strongly disagree<br>○ Slightly agree<br>○ Moderately agree<br>○ Quite agree<br>○ Strongly agree
D-24 The characteristics of my current fatigue remind me of the fatigue during the acute phase of the infection<br>*This question is only in the PCC questionnaire.	○ Strongly disagree<br>○ Slightly agree<br>○ Moderately agree<br>○ Quite agree<br>○ Strongly agree
D-25 The feeling of tiredness is accompanied by headaches	○ Strongly disagree<br>○ Slightly agree<br>○ Moderately agree<br>○ Quite agree<br>○ Strongly agree
D-26 The feeling of tiredness is accompanied by joint pain	○ Strongly disagree<br>○ Slightly agree<br>○ Moderately agree<br>○ Quite agree<br>○ Strongly agree
D-27 The feeling of tiredness disappears after resting	○ Strongly disagree<br>○ Slightly agree<br>○ Moderately agree<br>○ Quite agree<br>○ Strongly agree
D-28 I am unable to dose physical activity to predict fatigue episodes	○ Strongly disagree<br>○ Slightly agree<br>○ Moderately agree<br>○ Quite agree<br>○ Strongly agree
D-29 I am unable to dose cognitive activity to predict fatigue episodes	○ Strongly disagree<br>○ Slightly agree<br>○ Moderately agree<br>○ Quite agree<br>○ Strongly agree
D-30 I have "flare-ups" of worsening fatigue (flare-up: an episode of at least 24 hours of significant worsening of fatigue)	○ Strongly disagree<br>○ Slightly agree<br>○ Moderately agree<br>○ Quite agree<br>○ Strongly agree
D-31 I know what triggers these flare-ups	○ Strongly disagree<br>○ Slightly agree<br>○ Moderately agree<br>○ Quite agree<br>○ Strongly agree
D-32 Activities that motivate me generate less fatigue	○ Strongly disagree<br>○ Slightly agree<br>○ Moderately agree<br>○ Quite agree<br>○ Strongly agree

Scale E. Questionnaire for patients with fibromyalgia

Section for participants diagnosed with fibromyalgia	
Items	Options/response
E-1 Please specify the disease you have been diagnosed with by your doctor	○ Fibromyalgia<br>○ Chronic Fatigue Syndrome/Myalgic Encephalomyelitis
E-2 Date of diagnosis of fibromyalgia or myalgic encephalomyelitis/chronic fatigue syndrome (Example: January 7, 2019)	Options/response
E-3 Date of onset of symptoms of the disease (Example: January 7, 2019)	Options/response
E-4 Was the diagnosis of this disease (fibromyalgia or myalgic encephalomyelitis/chronic fatigue syndrome) related to any infection? If yes, please specify which one. If no, please answer 'no'	Options/response
E-5 Are you taking any treatment for fibromyalgia or myalgic encephalomyelitis/chronic fatigue syndrome? If yes, please specify which one. If no, please answer 'no'.	Options/response
E-6 If you have been infected with COVID-19, has the COVID-19 infection affected your fatigue? If yes, please specify how your fatigue changed and the duration of this change, or if the change is ongoing. If no, please answer 'no'	Options/response

Scale F. Questionnaire for patients with multiple sclerosis

Section for participants diagnosed with (MS)	
Items	Options/response
F-1 Do you know the type of Multiple Sclerosis you have?	○ Relapsing-Remitting Multiple Sclerosis (RRMS)<br>○ Secondary Progressive Multiple Sclerosis (SPMS)<br>○ Primary Progressive Multiple Sclerosis (PPMS)<br>○ I do not know the type of Multiple Sclerosis I have
F-2 Date of Multiple Sclerosis (MS) diagnosis (Example: January 7, 2019)	Options/response
F-3 Date of onset of symptoms of the disease (Example: January 7, 2019)	Options/response
F-4 Was the diagnosis of this disease (Multiple Sclerosis (MS)) related to any infection? If yes, please specify which one. If no, please answer 'no'.	Options/response
F-5 When was your last relapse?	Options/response
F-6 Are you taking any treatment for Multiple Sclerosis (MS)? If yes, please specify which one. If no, please answer 'no'	Options/response
F-7 If you have been infected with COVID-19, has the COVID-19 infection affected your fatigue? If yes, please specify how your fatigue changed and the duration of this change, or if the change is ongoing. If no, please answer 'no'	Options/response

Supplementary Table S1. Intensity Scale, adjusted for age as a covariate

	COVARIATE AGE			
	ANCOVA	Post-hoc analysis		
Item	Difference between groups F (P)	PCC vs Fibromyalgia	PCC vs MS	Fibromyalgia vs MS
C-1 I feel more tired	**9.626 (<.001)**	.979	**<.001**	**.001**
C-2 I need to rest more hours	**13.931 (<.001)**	.985	**<.001**	**<.001**
C-3 I have the sensation of lacking energy	**19.036 (<.001)**	.941	**<.001**	**<.001**
C-4 I have lost interest in activities I used to do	5.594 (.004)	.494	.023	.004
C-5 I feel like I have less muscle strength	**13.706 (<.001)**	.384	**<.001**	**.002**
C-6 I feel pain in my limbs	**41.250 (<.001)**	.029	**<.001**	**<.001**
C-7 Pain worsens my feeling of tiredness	**21.610 (<.001)**	.157	**<.001**	**<.001**
C-8 I feel like I have difficulty concentrating	**19.753 (<.001)**	.832	**<.001**	**<.001**
C-9 I feel like I don't feel like doing anything	**6.318 (.002)**	.013	.485	**.002**
C-10 I have trouble starting things because I feel like I won't be able to do them	3.380 (.035)	.212	.357	.027
C-11 I have trouble finishing things because I feel like I won't be able to finish them	2.594 (.076)	.208	.620	.070
C-12 I feel that my tiredness is permanent	**19.065 (<.001)**	.918	**<.001**	**<.001**
C-13 I feel tired regardless of the physical activity I do	**9.322 (<.001)**	.235	.003	**<.001**
C-14 Tiredness prevents me from doing physical exercise/activity	**19.798 (<.001)**	.627	**<.001**	**<.001**
C-15 I feel that tiredness has changed my life	**15.537 (<.001)**	.071	**<.001**	.009
C-16 Even though I feel low-spirited, I don't feel that tiredness is due to this; instead, I feel bad because I am tired	**10.900 (<.001)**	.160	**<.001**	.034
C-17 I notice that I am much more tired than my family or friends who also had COVID-19	**6.102 (.002)**	.245	.037	**.002**
C-18 The feeling of tiredness interferes with my sexual desire	**6.365 (.002)**	.608	.008	**.002**

Values are shown as mean (SD). Results that survived Bonferroni correction are highlighted in bold. PCC = Post-COVID Condition; MS = multiple sclerosis.
